# Supplementary material for: Deep Sequencing Reveals Central Nervous System Compartmentalization in Multiple Transmitted/Founder Virus Acute HIV-1 Infection
Source: Cells. 2019 Aug 15;8(8):902. doi: 10.3390/cells8080902 (PMC6721674; doi:10.3390/cells8080902)
Supplement: Supplementary file 1 [file cells-08-00902-s001.pdf]

Table S1. Primers used for cDNA synthesis and SGA amplification of full and half genomes, and envelope genes

Primer	Amplicon	Name	Sequence (5′ → 3′)
cDNA primers	Full genome, 3′-half genome, and envelope	Oligo(dT)	$T_{(20)}$
	5′-half genome	JL68RV2	CTTCTTCCTGCCATAGGAGATGCCTAAG
SGA Full Genome (HXB2 789-9496)	1st Round	MSF12B	AAATCTCTAGCAGTGGCGCCCGAACAG
		UNINEF-7’	GCACTCAAGGCAAGCTTTATTGAGGCTT
	2nd Round	GAG763	TGACTAGCGGAGGCTAGAAGGAGAGA
		TATANEF	GCAGCTGCTTATATGCAGGATCTGAGGG
SGA 5′ half-genome (HXB2 789-5852)	1st Round	MSF12B	AAATCTCTAGCAGTGGCGCCCGAACAG
		JL68RV2	CTTCTTCCTGCCATAGGAGATGCCTAAG
	2nd Round	GAG763	TGACTAGCGGAGGCTAGAAGGAGAGA
		TATAD’	TTCCCGGRTGKTTCCAGGGCTCTA
SGA 3′ half-genome (HXB2 (4559-9496)	1st Round	POLJV2	GAAGCYATGCATGGACAAGTRGA
		UNINEF-7’	GCACTCAAGGCAAGCTTTATTGAGGCTT
	2nd Round	POLK3	TAAARYTAGCAGGAAGATGGCCAGT
		TATANEF	GCAGCTGCTTATATGCAGGATCTGAGGG

Table S2. Primers used for targeted deep sequencing

POL Region (HXB2 1817-3520)				
**Primer**	**Amplicon**	**Type**	**Name**	**Sequence (5′ → 3′)**
Universal Primers	1st Round	Outer	Pol outer primer forward	TAGAAGAAATGATGACAGC
		Outer	Pol outer primer reverse	TCTTTTGATGGGTCATA
	PRO	Inner	Pro_F	CAGGCTAATTTTTTAGGGA
		Inner	Pro_R	CATCCATTCCTGGCTTTA
	PolSegment	Inner	Pol seg1 forward	TAAAGCCAGGAATGGATGG
		Inner	Pol seg2 reverse	TGTATATCATTGACAGTCC
Specific Primers	1st Round	Outer	PS2Rv3	TFCCCAATTTAGTTTTCC
		Outer	Pol out R288	CCTGTGCATGGAGTATATTATGA
		Outer	Pol out F019	TAGAGGAAATGATGACAGC
		Outer	Pol out Rv4	TGCCAATTCTAATTCTGCTT
	PRO	Inner	PRO_F024	CAAATGAAAGACTGCACTGA
		Inner	Pro_R034	AAAGTACAACCAATTTGGGT
		Inner	Pro_R023	TCTAACCTTTGGCCCATC
		Inner	Pro_Fv3	CTAGAAAAAAGGGTTGTTGG
		Inner	Pro_Rv2	AACCTTTGGTCCATCCA
		Inner	ProFv2	AAATGTGGAAAGGAAGGAC
	PolSegment	Inner	PS1_F036	AAGCCAGGAATGGAT
		Inner	PS2Rv4	TGTATATCATTGACAGTCCAGC
		Inner	PS1_F034	AGTAGGACCTACACCTGTCA
		Inner	PS2_R004	TGTATATCATTGACAGTCC
		Inner	PS2R288	AGCTCCATCCTGACAAATG
		Inner	PS2Rv2	AGTTCTATTGGCTGGAC
		Inner	PS2Rv3	TGCCCAATTTAGTTTTCC

ENV V2 Region (HXB2 6499-7034)				
**Primer**	**Amplicon**	**Type**	**Name**	**Sequence (5′ → 3′)**
Universal Primers	1st Round	Outer	v2 env outer forward	TAATTCCATGTGTGCATTG
		Outer	v2 env outer reverse	TTATGGGATCAAAGTCTA
	2nd Round	Inner	V2outFext	ATTTTAACATGTGGAAAAATAACATG<br>GTAGAACAGATGCAAGAGGATGTAAT<br>CAGTTTATGGGATCAAAGTCTA
		Inner	V2outRext	TATCTCATCTTCTGCTACACTCCCATT<br>TAACACCAATTGAGTTCATACCACTCC<br>CTTAATTCCATGTGTGCATGG
Specific Primers	1st and 2nd Round	Outer/Inner	v2_inF036	ACAGACCCCAACCCA
		Outer/Inner	v2 out Rv2	TAATTCCATGTGTGCATTGT
		Outer/Inner	v2_out_F036	ATGTAATCAGTTTATGGGA
		Outer/Inner	V2_out_F016	TTATGGGATCAAAGCCTA
		Outer/Inner	V2 out F035 Pacbio	TACCCACAGACCCCAA

		Outer/Inner	v2 in Fv3	TTTAACATGTGGAAAAATAA
		Outer/Inner	V2_OUT_F_v3	AAAFCACATGTGACAGAAGT
		Outer/Inner	v2 out R036 ext	CATTAAGGTGCACTATTATGGTTT
		Outer	034_V2_F01	GCACAATGTCTGGGCCA
		Outer	034_V2_F02	GACCCCAACCCACAAGAAA
		Inner	034_V2_R01	TATTCCATGTGTGCATTGTACTGA
		Inner	034_V2_R02	TGACATTTTTACATGGCCCTGT

Table S3. Reproducibility in quantifying the proportions of HIV-1 variants by Ion Torrent and PacBio next generation sequencing platforms

Sample	Region	Rep	Variant 1	Variant 2	Variant 3	Variant 4	Variant 5
Sample 1 (Ion Torrent)	POL PR I	1	69.50	15.56	3.41	3.07	2.49
		2	65.47	16.00	3.53	3.86	3.33
		3	67.29	14.70	3.81	3.70	3.31
		**CV**	**2.99%**	**4.29%**	**5.70%**	**11.81%**	**15.76%**
	POL RT I	1	77.90	12.12	8.31	1.67	
		2	77.03	13.16	7.81	2.01	
		3	77.90	12.56	7.83	1.71	
		**CV**	**0.65%**	**4.13%**	**3.54%**	**10.23%**	

Sample	Region	Rep	Variant 1	Variant 2	Variant 3	Variant 4	Variant 5
Sample 2 (Ion Torrent)	POL PR I	1	71.82	19.46	3.71	3.38	1.63
		2	71.04	19.94	3.91	3.79	1.32
		3	72.69	18.82	3.87	3.21	1.42
		**CV**	**1.15%**	**2.89%**	**2.78%**	**8.69%**	**10.95%**
	POL PR II	1	76.44	22.43	1.13		
		2	75.78	22.93	1.29		
		3	77.09	21.93	0.99		
		**CV**	**0.86%**	**2.26%**	**13.13%**		
	POL RT I	1	74.03	22.73	2.02		
		2	75.18	21.93	2.02		
		3	74.13	23.15	2.05		
		**CV**	**1.09%**	**2.73%**	**0.92%**		
	POL RT II	1	73.16	25.17	1.67		
		2	73.63	22.71	2.82		
		3	72.03	24.18	3.02		
		**CV**	**1.13%**	**5.16%**	**29.02%**		

Sample	Region	Rep	Variant 1	Variant 2	Variant 3	Variant 4	Variant 5
Sample 3 (Ion Torrent)	POL PR I	1	37.86	48.97	6.37	5.59	1.20
		2	37.22	50.27	6.02	5.37	1.11
		3	37.41	50.08	6.16	5.30	1.05
		**CV**	**0.87%**	**1.41%**	**2.84%**	**2.83%**	**6.93%**
	POL PR II	1	50.34	48.95	0.70		
		2	49.85	49.38	0.77		
		3	50.83	48.49	0.68		
		**CV**	**0.98%**	**0.91%**	**6.92%**		
	POL RT I	1	49.06	50.94			
		2	49.42	49.97			
		3	49.95	50.05			
		**CV**	**0.91%**	**1.07%**			
	POL RT II	1	48.04	51.96			
		2	41.11	58.89			
		3	49.90	50.10			
		**CV**	**10.00%**	**8.64%**			

Sample	Region	Rep	Variant 1	Variant 2	"others"
Sample 4 (PacBio)	ENV V2	1	76.30	23.52	0.18
		2	73.48	26.25	0.27
		3	74.18	25.5	0.32
		**CV**	**1.97%**	**5.62%**	**27.21%**

Figure S2

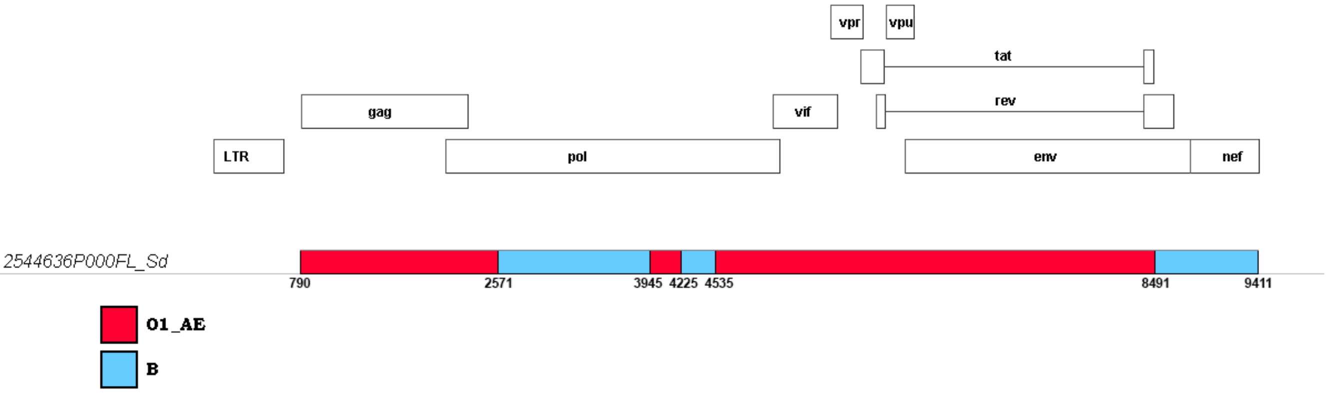

vpr
vpu
tat
gag
vif
rev
LTR
pol
env
nef
2544636P000FL_Sd
790
2571
3945
4225
4535
8491
9411
01_AE
B

Figure S1a
5′ Half genome

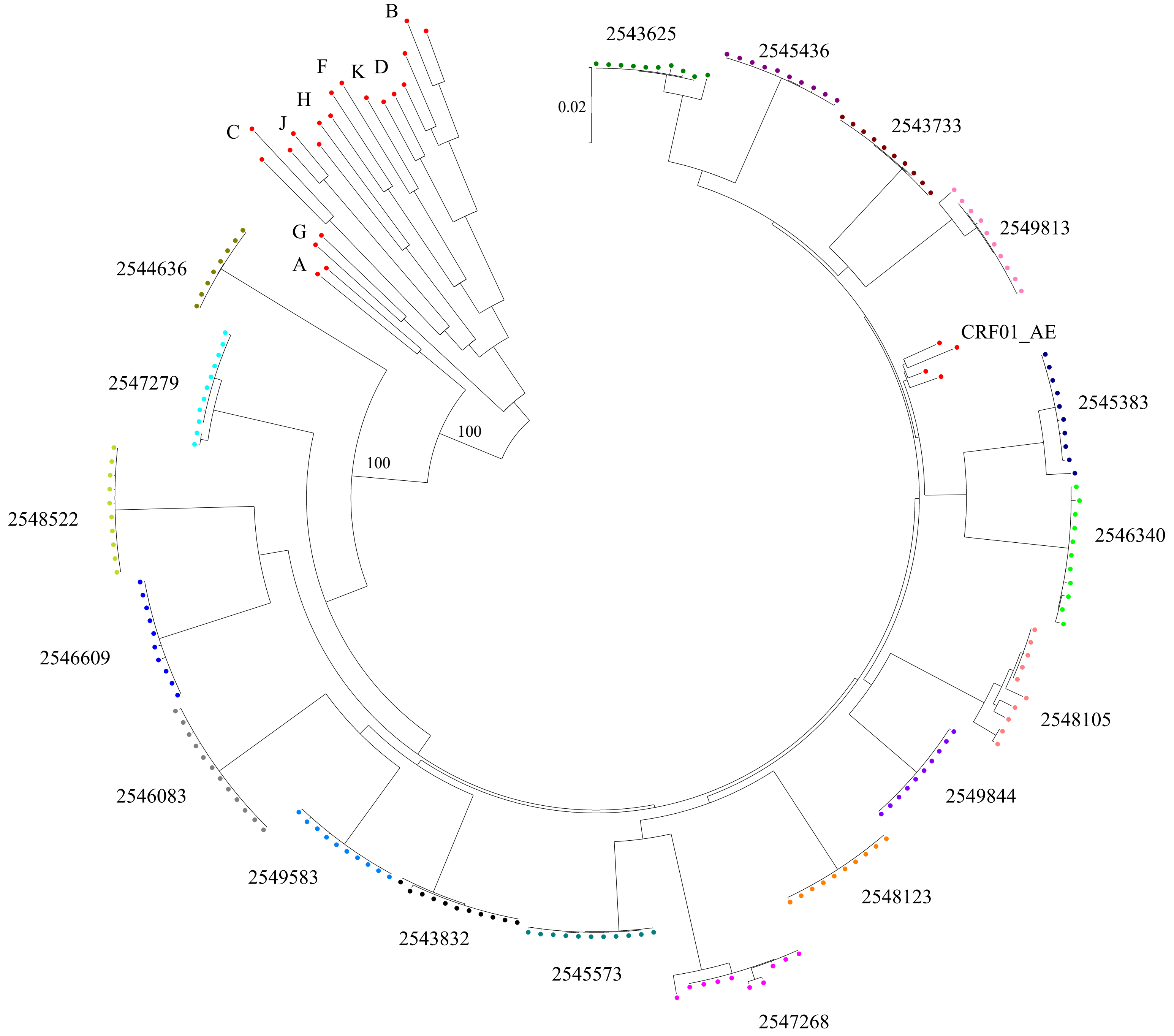

Figure S1b
3′ Half genome

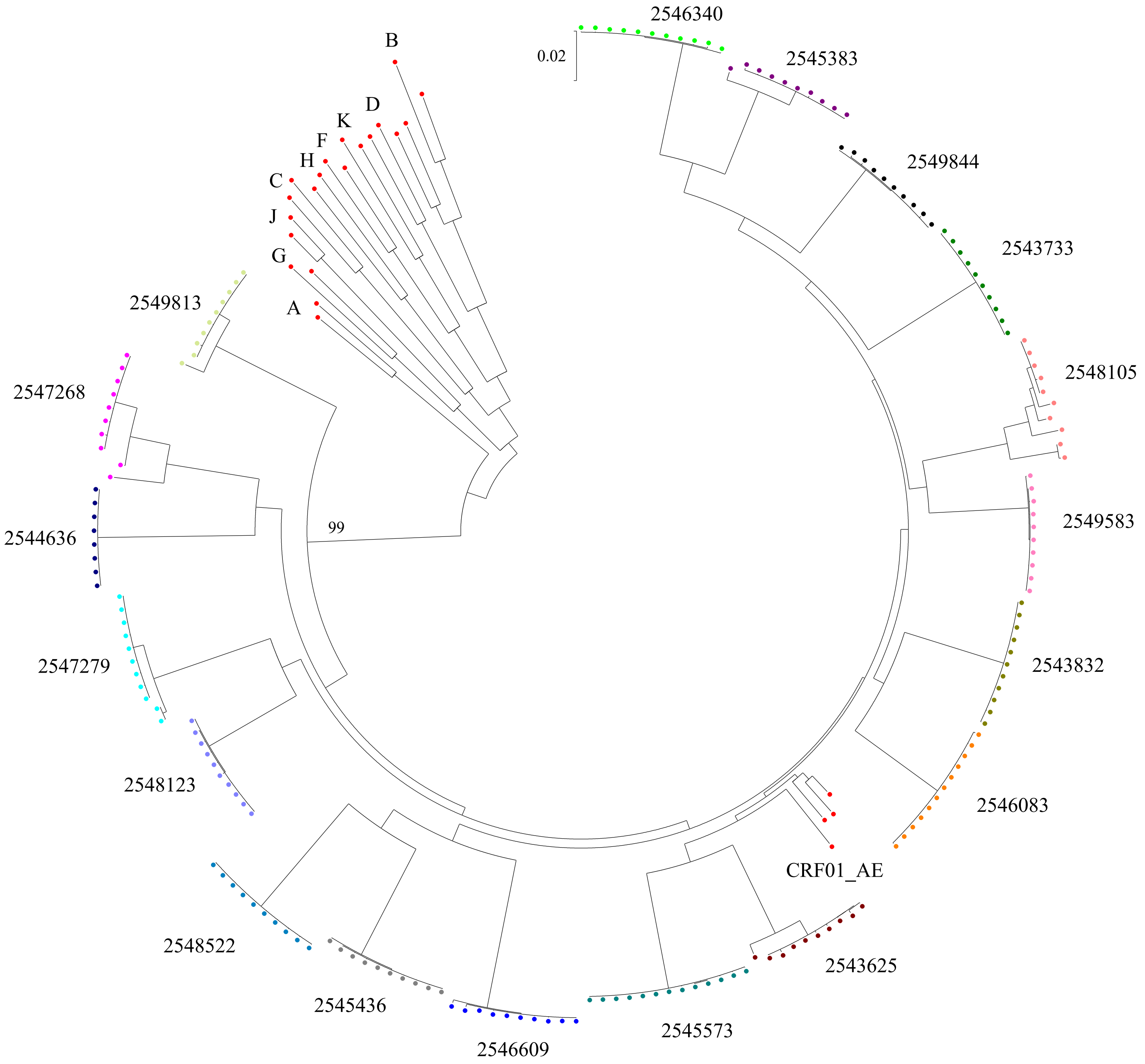

Figure S3 a

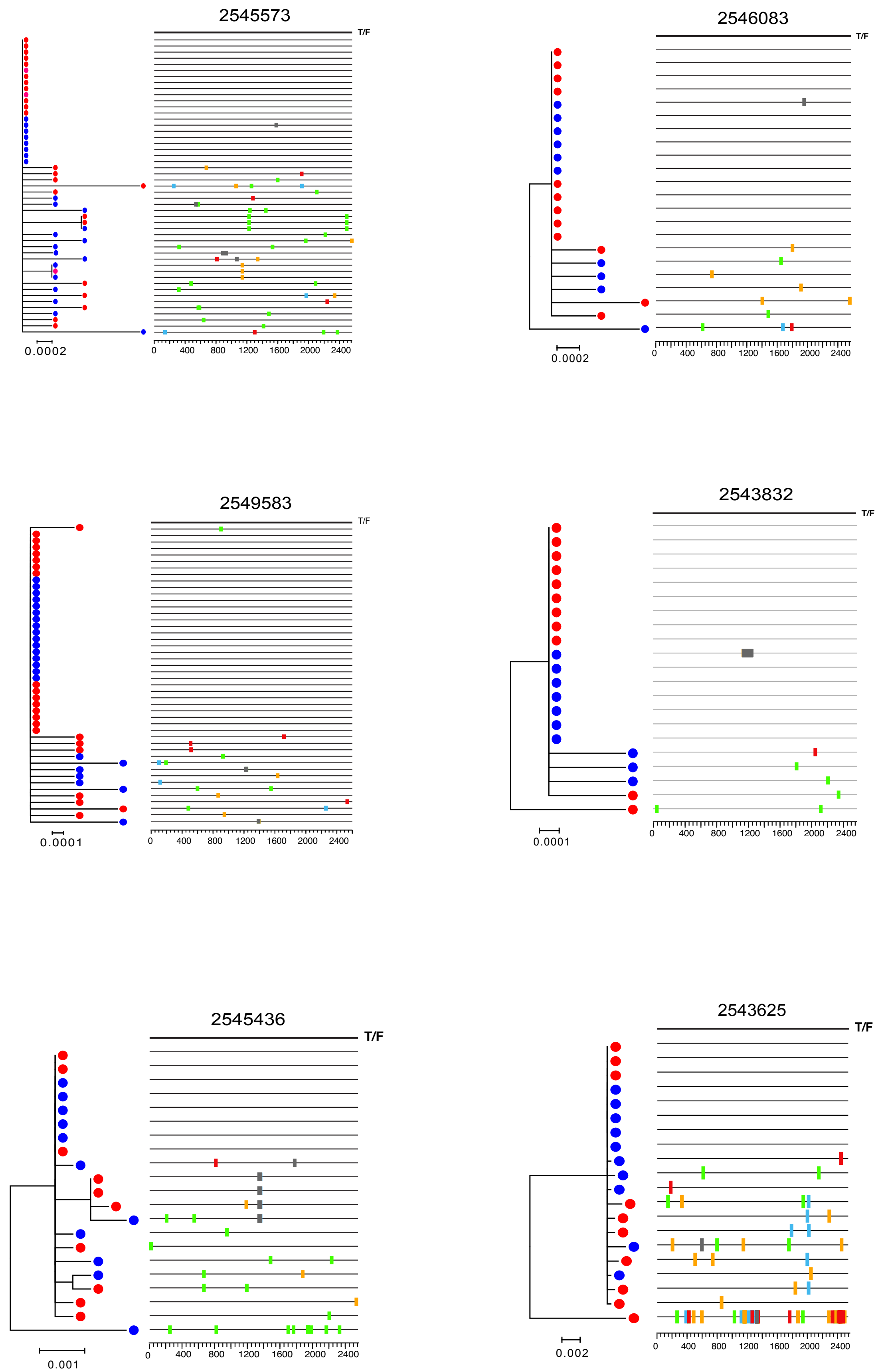
2545573
T/F
0.0002
0 400 800 1200 1600 2000 2400
2546083
T/F
0.0002
0 400 800 1200 1600 2000 2400
2549583
T/F
0.0001
0 400 800 1200 1600 2000 2400
2543832
T/F
0.0001
0 400 800 1200 1600 2000 2400
2545436
T/F
0.001
0 400 800 1200 1600 2000 2400
2543625
T/F
0.002
0 400 800 1200 1600 2000 2400

Figure S3 b

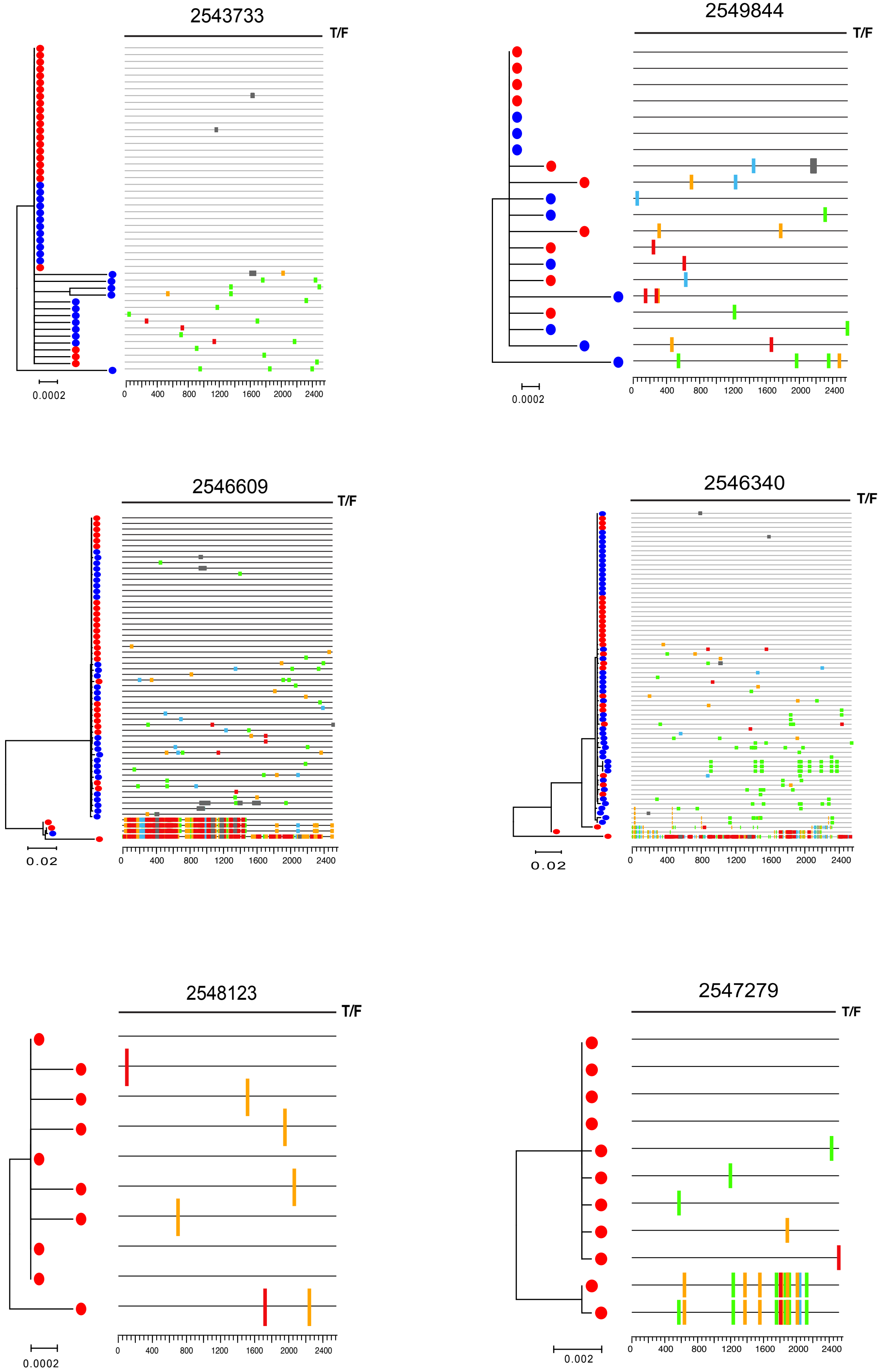
2543733
T/F
0.0002
0 400 800 1200 1600 2000 2400
2549844
T/F
0.0002
0 400 800 1200 1600 2000 2400
2546609
T/F
0.02
0 400 800 1200 1600 2000 2400
2546340
T/F
0.02
0 400 800 1200 1600 2000 2400
2548123
T/F
0.0002
0 400 800 1200 1600 2000 2400
2547279
T/F
0.002
0 400 800 1200 1600 2000 2400

Figure S3 c

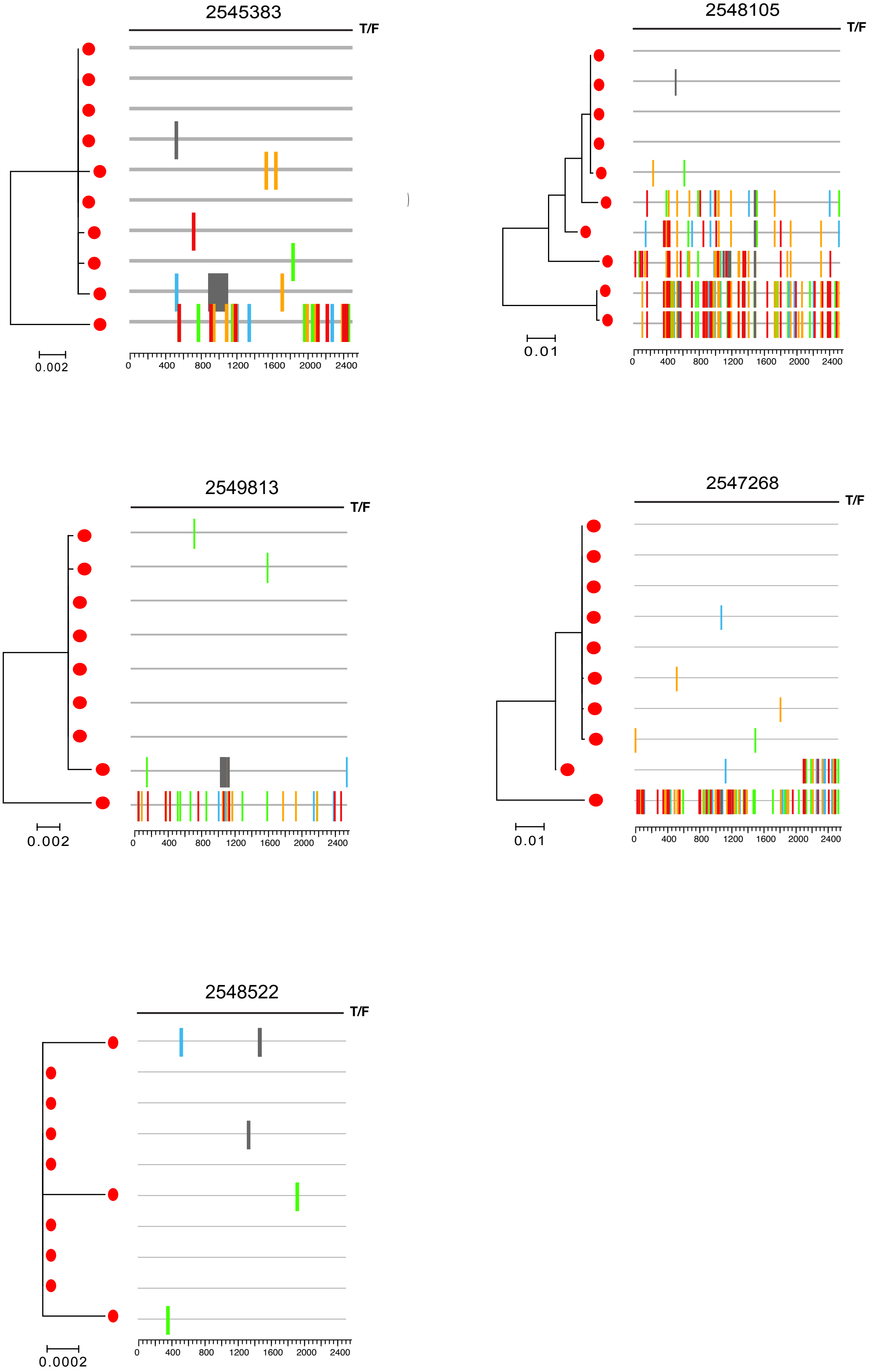
2545383
T/F
0.002
0
400
800
1200
1600
2000
2400
2548105
T/F
0.01
0
400
800
1200
1600
2000
2400
2549813
T/F
0.002
0
400
800
1200
1600
2000
2400
2547268
T/F
0.01
0
400
800
1200
1600
2000
2400
2548522
T/F
0.0002
0
400
800
1200
1600
2000
2400

Figure S4

Proportions of plasma and CSF variants from five additional multiple T/F participants observed by next generation sequencing platforms. In the regions of the genome studied by Ion Torrent, a locus was determined within a window of 250 base pairs (bp) based on the reliable read length of sequences. Most of the regions sequenced using Ion Torrent had more than one locus identified, whereas only one locus was identified on PacBio derived sequences with a read length between 350 and 650bp. a) Participant 2546609, b) Participant 2545383, c) Participant 2548105, d) Participant 2543625, and e) Participant 2547268.

Figure S4a. Participant 2546609

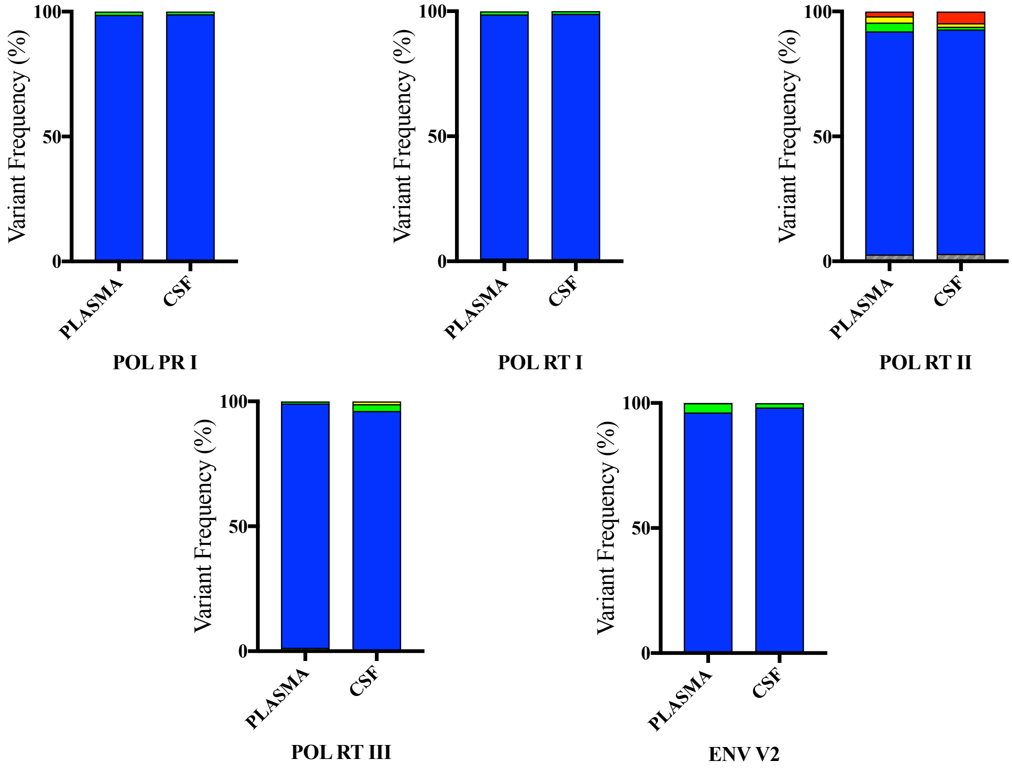

	POL PR I			POL RT I			POL RT II			POL RT III			ENV V2		
HXB2 Position	2126 - 2147			2578 - 2746			2854 - 2975			3068 - 3135			6573 - 6959		
Variant (%)	Plasma	CSF	Fold	Plasma	CSF	Fold	Plasma	CSF	Fold	Plasma	CSF	Fold	Plasma	CSF	Fold
1	98.72	98.25	1.00	97.58	97.99	1.00	89.42	89.80	1.00	97.76	96.14	1.02	96.18	98.24	1.02
2	1.28	1.04	1.23	1.26	1.03	1.22	3.49	1.08	3.23	0.90	2.76	3.07	3.77	1.68	2.24
3							2.44	1.43	1.71	-	1.10	NA			
4							1.97	4.71	2.39						
"others"	-	0.71		1.15	0.98		2.67	2.99		1.34	-		0.05	0.07	

Plasma and CSF were collected at 28 days and 29 days post estimated infection, respectively. Three genome regions were studied: POL PR, POL RT, and ENV V2. There was one locus identified in POL PR corresponding to HXB2 position 2126-2147 (POL PR I). Three loci were identified in POL RT corresponding to HXB2 position 2578-2746 (POL RT I), 2854-2975 (POL RT II), and 3068-3135 (POL RT III). One locus was identified in the ENV V2 region corresponding to HXB2 number 6573-6959. In all loci, there was no difference between compartments for the major variant with fold differences ranging from 1.00-1.02. There were no minor variants present (>3%) in both compartments.

Figure S4b. Participant 2545383

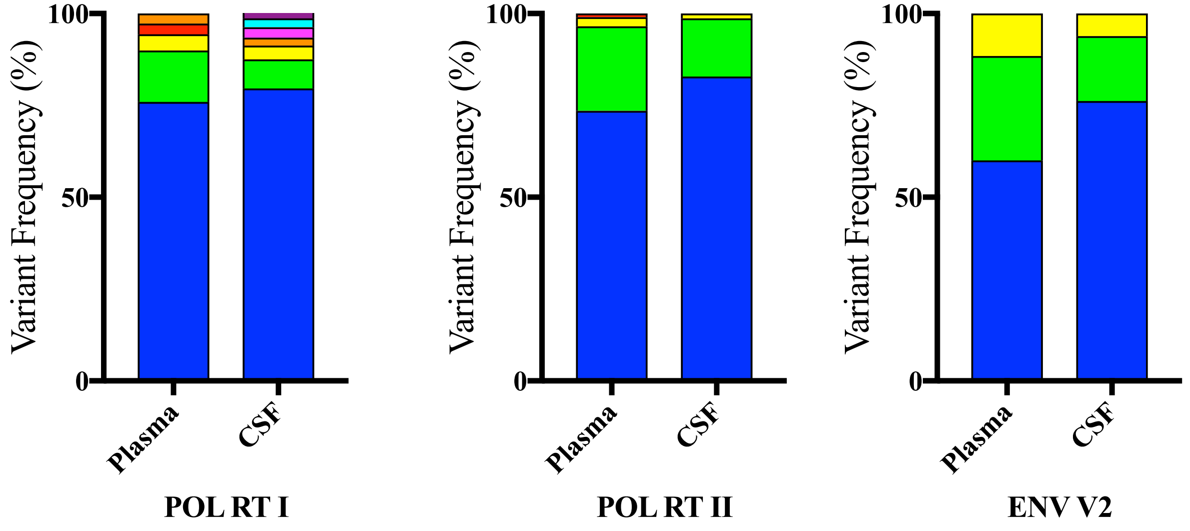

	POL RT I			POL RT II			ENV V2		
**HXB2 Position**	2647 - 2807			2873 - 2969			6573 - 6959		
**Variant (%)**	**Plasma**	**CSF**	**Fold**	**Plasma**	**CSF**	**Fold**	**Plasma**	**CSF**	**Fold**
1	75.95	79.63	1.05	73.58	82.86	1.13	59.29	75.53	1.27
2	14.04	7.87	1.78	22.96	15.81	1.45	28.43	17.71	1.61
3	4.35	3.76	1.16	2.46	1.33	1.85	11.54	6.09	1.89
4	2.91	-	NA	1.00	-	NA			
5	2.75	2.16	1.27						
6	-	2.90	NA						
7	-	2.37	NA						
8	-	1.31	NA						
"others"	-	-		-	-		0.74	0.67	

Plasma and CSF were collected at 20 days post estimated infection. Genome regions POL RT and ENV V2 were analyzed. Data for POL PR was omitted from the analysis due to the quality of the sequences from both compartments failing the criteria that an equal number of forward and reverse sequences be obtained. In POL RT corresponding to HXB2 position 2647-2807 (POL RT I) and 2873-2969 (POL RT II), the proportions of major variants identified were not different. However, the minor variants were lower in CSF than in plasma with the fold differences ranging from 1.16 to 1.78. Similar findings to POL RT were also observed in ENV V2 region corresponding to HXB2 position 6573-6959. The major variant was slightly higher in CSF with a fold difference of 1.27, whereas the proportions of two minor variants identified were lower in CSF with fold differences of 1.61 and 1.89.

Figure S4c. Participant 2548105

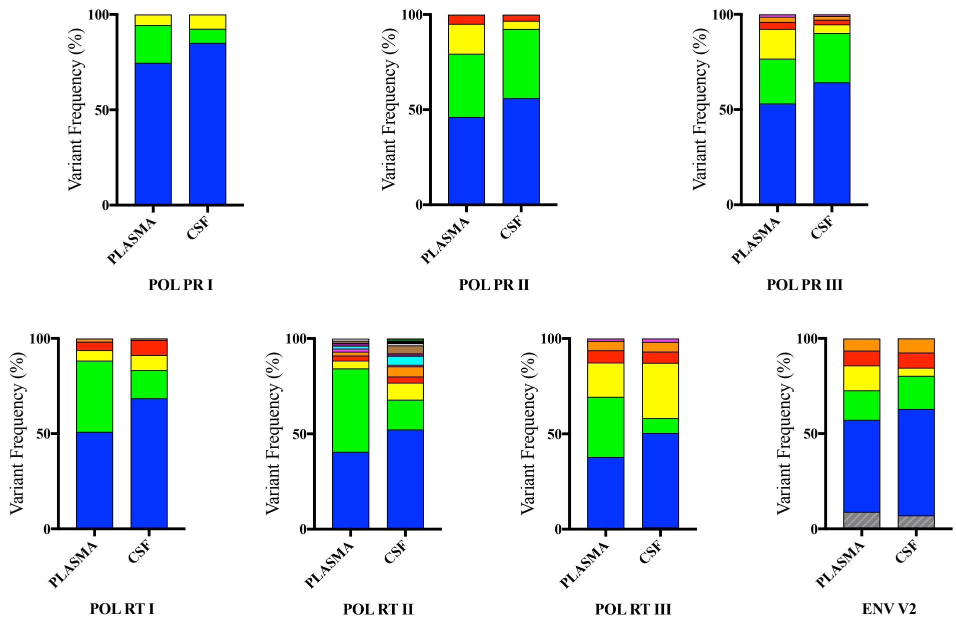

	POL PR I			POL PR II			POL PR III			POL RT I			POL RT II			POL RT III			ENV V2		
HXB2 Position	2124-2139			2205-2372			2477-2543			2750-2849			2885-3032			3066-3191			6522-7067		
Variant (%)	Plasma	CSF	Fold	Plasma	CSF	Fold	Plasma	CSF	Fold	Plasma	CSF	Fold	Plasma	CSF	Fold	Plasma	CSF	Fold	Plasma	CSF	Fold
1	74.71	84.46	1.13	46.13	56.12	1.22	53.28	64.37	1.21	50.29	68.62	1.36	40.60	51.51	1.27	37.85	49.55	1.31	48.34	55.77	1.15
2	19.72	7.40	2.66	33.35	36.38	1.09	23.55	25.83	1.10	37.45	14.77	2.54	43.80	15.58	2.81	31.62	7.83	4.04	15.49	17.44	1.13
3	5.57	7.47	1.34	15.69	4.23	3.71	15.55	4.63	3.36	5.46	7.87	1.44	4.06	8.92	2.20	18.02	29.01	1.61	13.07	4.29	3.05
4				4.83	3.27	1.48	3.63	2.35	1.54	4.47	7.93	1.77	2.63	3.31	1.26	6.36	5.84	1.09	7.76	8.00	1.03
5							2.77	1.98	1.40	1.68	0.81	2.07	2.00	5.27	2.64	4.98	5.09	1.02	6.30	7.35	1.17
6							1.22	0.84	1.45				1.69	0.89	1.90	1.17	1.74	1.49			
7													1.52	4.65	3.06						
8													1.39	1.27	1.09						
9													1.16	4.21	3.63						
10													1.14	1.10	1.04						
11													-	1.23	NA						
12													-	1.23	NA						
"others"	-	0.67		-	-		-	-		0.64	-		-	0.83		-	0.94		8.99	7.15	

Plasma and CSF were collected at 25 days and 26 days post estimated infection, respectively. Genome regions POL PR, POL RT, and ENV V2 were studied. Three loci were identified in POL PR corresponding to HXB2 position 2124-2139 (POL PR I), 2205-2372 (POL PR II), and 2477-2543 (POL PR III). Significant differences in the proportions of some minor variants were observed between plasma and CSF, with some present in plasma at greater proportion than CSF and vice versa. The fold differences ranged from 2.66 to 3.71. The PRO RT region also had three loci identified corresponding to HXB2 position 2750-2849 (POL RT I), 2885-3032 (POL RT II), and 3066-3191 (POL PR III). The proportions of major variants between two compartments in these loci were slightly different, with fold differences ranging from 1.27 to 1.36. The minor variants in these 3 loci had fold differences ranging from 1.44 to 4.04, and the proportion of some variants increased while others decreased. In the ENV V2 region corresponding to HXB2 position 6522-7067, one of the minor variants identified was present at a frequency 3-fold lower in CSF than in plasma.

Figure S4d. Participant 2543625

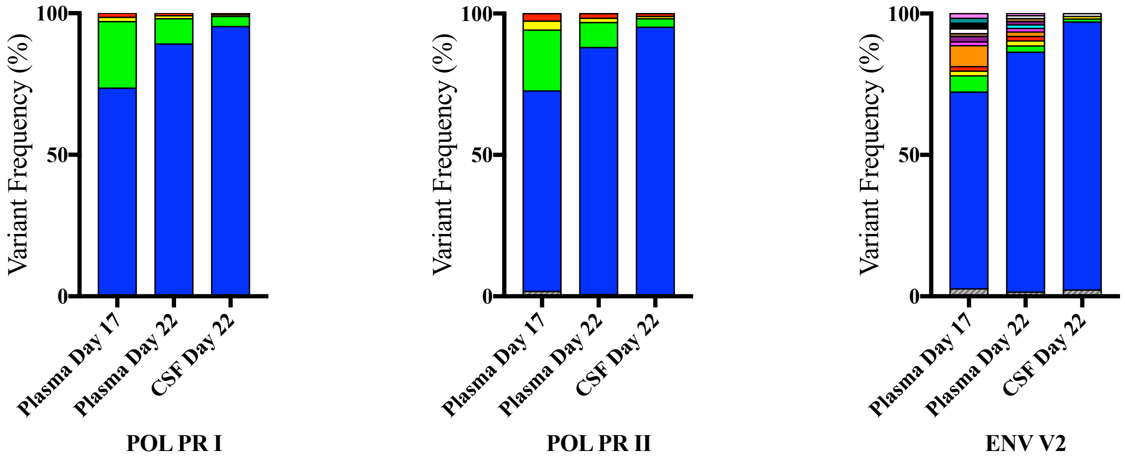

	POL PR I				POL PR II				ENV V2			
HXB2 Position	2184 - 2207				2339 - 2441				6573 - 6959			
Variant (%)	Plasma (Day 17)	Plasma (Day 22)	CSF (Day 22)	Fold	Plasma (Day 17)	Plasma (Day 22)	CSF (Day 22)	Fold	Plasma (Day 17)	Plasma (Day 22)	CSF (Day 22)	Fold
1	73.53	89.15	95.27	1.07	70.88	87.40	94.59	1.08	69.52	84.84	94.74	1.12
2	23.47	8.88	3.63	2.45	21.53	8.78	3.00	2.93	5.83	2.28	1.13	2.02
3	1.53	1.07	0.54	1.98	3.19	1.51	0.90	1.68	1.62	1.74	0.90	1.93
4	1.47	0.90	0.56	1.61	2.69	1.72	0.99	1.74	1.60	1.60	-	NA
5									7.39	1.54	-	NA
6									1.33	1.29	-	NA
7									-	1.20	-	NA
8									1.93	1.18	-	NA
9									1.05	1.10	-	NA
10									1.64	1.03	1.01	1.02
11									1.97	-	-	NA
12									1.75	-	-	NA
13									1.72	0.78	-	NA
"others"	-	-	-		1.71	0.60	0.51		2.64	1.44	2.22	

Plasma samples were collected at 17 and 22 days post estimated infection, and CSF was collected at day 22 post estimated infection. Hence, we were able to explore the dynamics of HIV-1 variants in plasma from day 17 and day 22, and compare proportions of HIV-1 variants between two compartments at day 22. Only genome regions POL PR and ENV V2 were analyzed in this participant due to insufficient sequencing data in POL RT. There were two loci identified in POL PR: POL PR I (HXB2 position 2184-2207) and POL PR II (2339-2441). We observed changes in virus proportions between plasma day 17 and day 22 in both regions. The proportion of the major variant increased between the two collections while the proportion of the minor variant decreased. The proportions of major variants in plasma and CSF were not different at day 22, but fold difference increase in the proportions of minor variants identified above 3% ranged from 2.45 to 2.93. In the ENV V2 region (6573-6959), no minor variants were detected above 3% at day 22. The major variant identified in this region was only slightly increased in CSF compared to plasma with a fold difference of 1.12.

Figure S4e. Participant 2547268

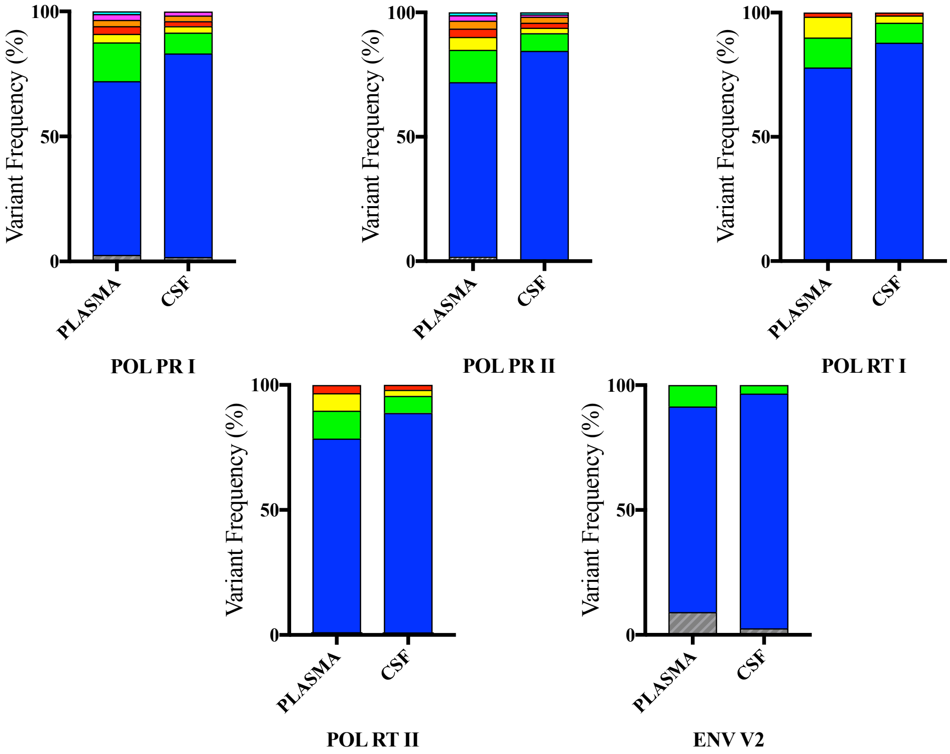

	POL PR I			POL PR II			POL RT I			POL RT II			ENV V2		
HXB2 Position	2076 - 2223			2435 - 2558			2780 - 2850			3188 - 3218			6419 - 6959		
Variant (%)	Plasma	CSF	Fold	Plasma	CSF	Fold	Plasma	CSF	Fold	Plasma	CSF	Fold	Plasma	CSF	Fold
1	69.50	81.43	1.17	70.16	83.94	1.20	77.90	87.90	1.13	77.85	88.27	1.13	82.34	93.96	1.14
2	15.56	8.32	1.87	13.00	7.10	1.83	12.12	8.02	1.51	11.17	6.80	1.64	8.56	3.38	2.53
3	3.41	2.60	1.31	5.20	2.15	2.42	8.31	2.91	2.86	6.97	2.42	2.88			
4	3.07	2.01	1.53	3.32	2.09	1.59	1.67	1.17	1.43	3.31	1.98	1.67			
5	2.49	2.21	1.13	3.25	2.38	1.37									
6	2.34	1.62	1.44	2.10	0.93	2.26									
7	1.03	-	NA	1.17	0.81	1.44									
"others"	2.60	1.80		1.80	0.61		-	-		0.69	0.53		9.10	2.66	

Plasma and CSF were collected at 23 days and 24 days post estimated infection, respectively. There were three studied genome regions, POL PR, POL RT, and ENV V2. There were two loci identified in POL PR corresponding to HXB2 position 2076-2223 (POL PR I) and 2435-2558 (POL PR II). In both loci, the major variants were present in CSF at slightly higher proportions of 1.17 and 1.20-fold difference compared to plasma. Meanwhile the minor variants above 3% were higher in plasma than CSF with fold differences of 1.87 and 1.83. In POL RT, two loci were identified corresponding to HXB2 number 2780-2850 and 3188-3218. The major variants were present in CSF at slightly higher proportions of 1.13-fold difference compared to plasma. The minor variants were present significantly lower in CSF in both loci with fold differences of 1.51 and 1.64. Similar findings were identified in ENV V2 region corresponding to HXB2 number 6419-6959. The major variant presented at 1.14-fold higher in CSF and minor variant presented at 2.53-fold higher in plasma.
